# Supplementary material for: Cross-tissue integration of genetic and epigenetic data offers insight into autism spectrum disorder
Source: Nat Commun. 2017 Oct 24;8:1011. doi: 10.1038/s41467-017-00868-y (PMC5654961; doi:10.1038/s41467-017-00868-y)
Supplement: Supplementary file 1 — Supplementary Information [file 41467_2017_868_MOESM1_ESM.pdf]

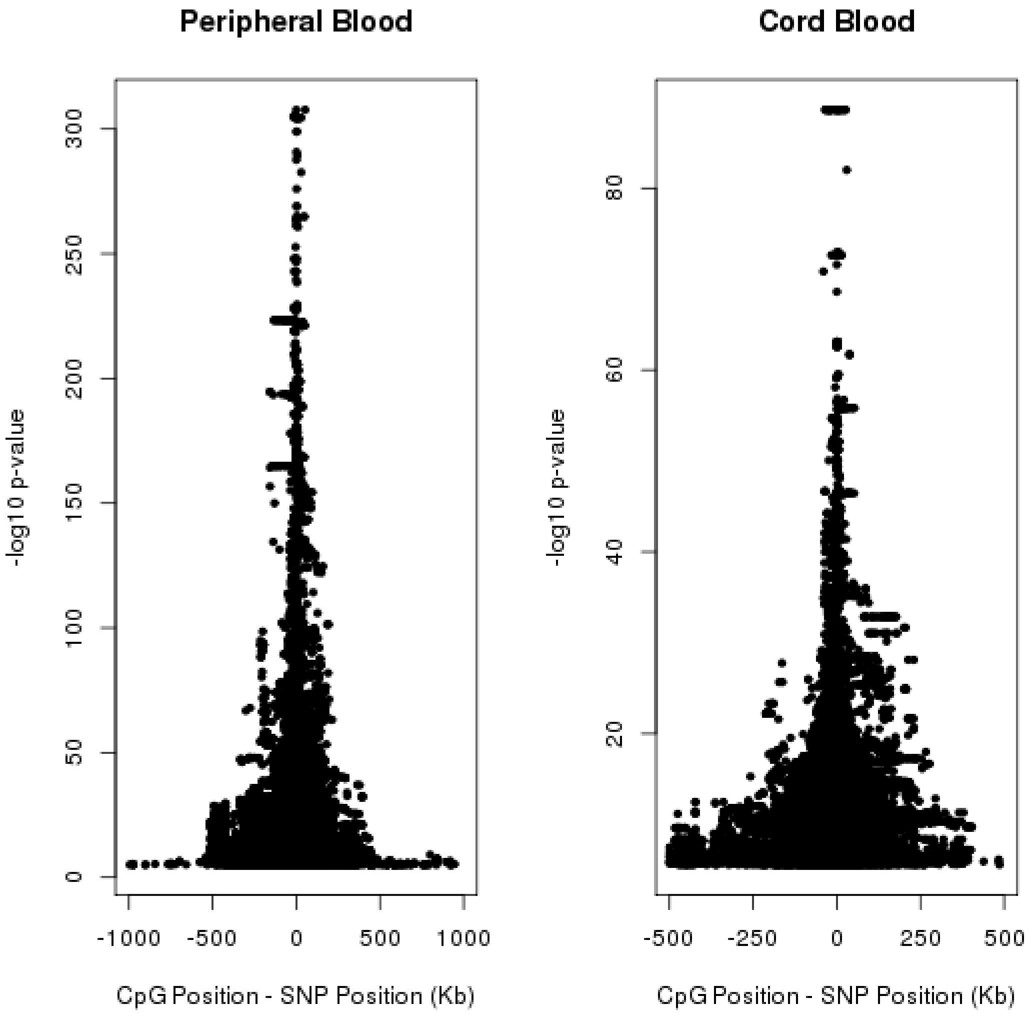

Supplementary Figure 1: **The relationship between degree of significance and distance between SNP and CpG site on chromosome 21.** Degree of significance (y-axis) defined by -log10 p-value. Only meQTLs present at FDR = 5% are shown. The degree of significance decays with increasing distance. Left panel shows relationship for SEED peripheral blood data and right panel shows relationship for EARLI cord blood data.

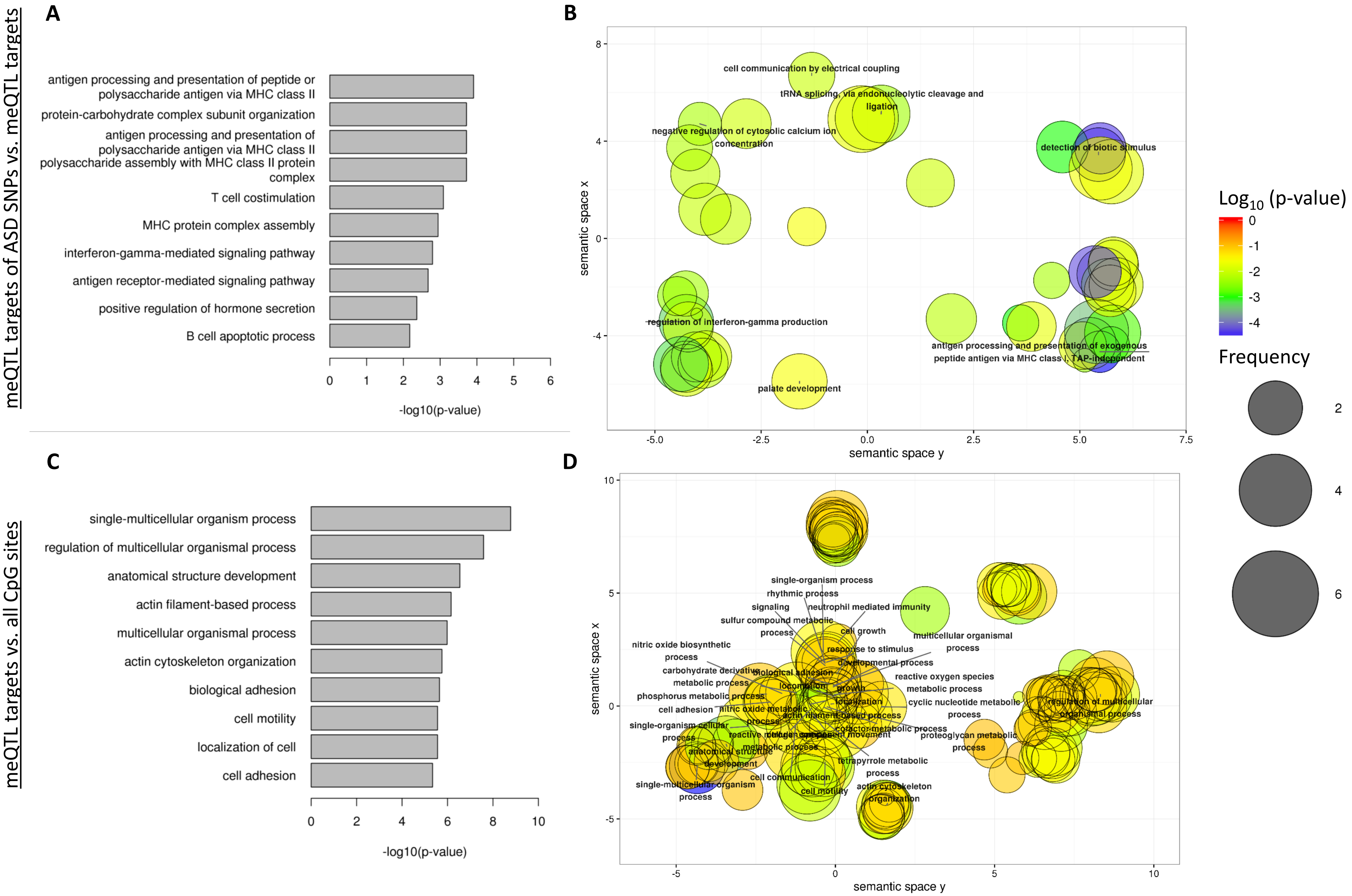

Supplementary Figure 2: **Gene Ontology enrichment analysis for meQTL targets in peripheral blood.** Gene Ontology (GO) enrichment analysis via the 'gometh' function in the *MissMethyl* R package. The results for the "biological processes" group were pruned to remove overlapping terms using the REVIGO software. Results shown for meQTL targets of ASD-related (PGC p-value < 1E-4) SNPs and their proxies (r^2) against a background of all meQTL targets (n = 201 vs n = 59,308; **Panels A+B**) and for meQTL targets against a background of all CpG sties tested (n = 59,308 vs n = 290,066; **Panels C+D**). meQTL targets all defined via meQTL p-value threshold = FDR 5%. **Panels A+C**) The top 10 biological process by GO enrichment p-value after REVIGO pruning. **Panel B+D**) A multi-dimensional scaling projection of the semantic similarity in nominally significant (enrichment p-value < 0.05) GO terms produced by REVIGO. Clusters are identified via labeling of the terms with both the least redundancy and highest degree of enrichment ('dispensability' value < 0.15). Color reflect degree of significance and increasing size reflects greater frequency of term in GO database.

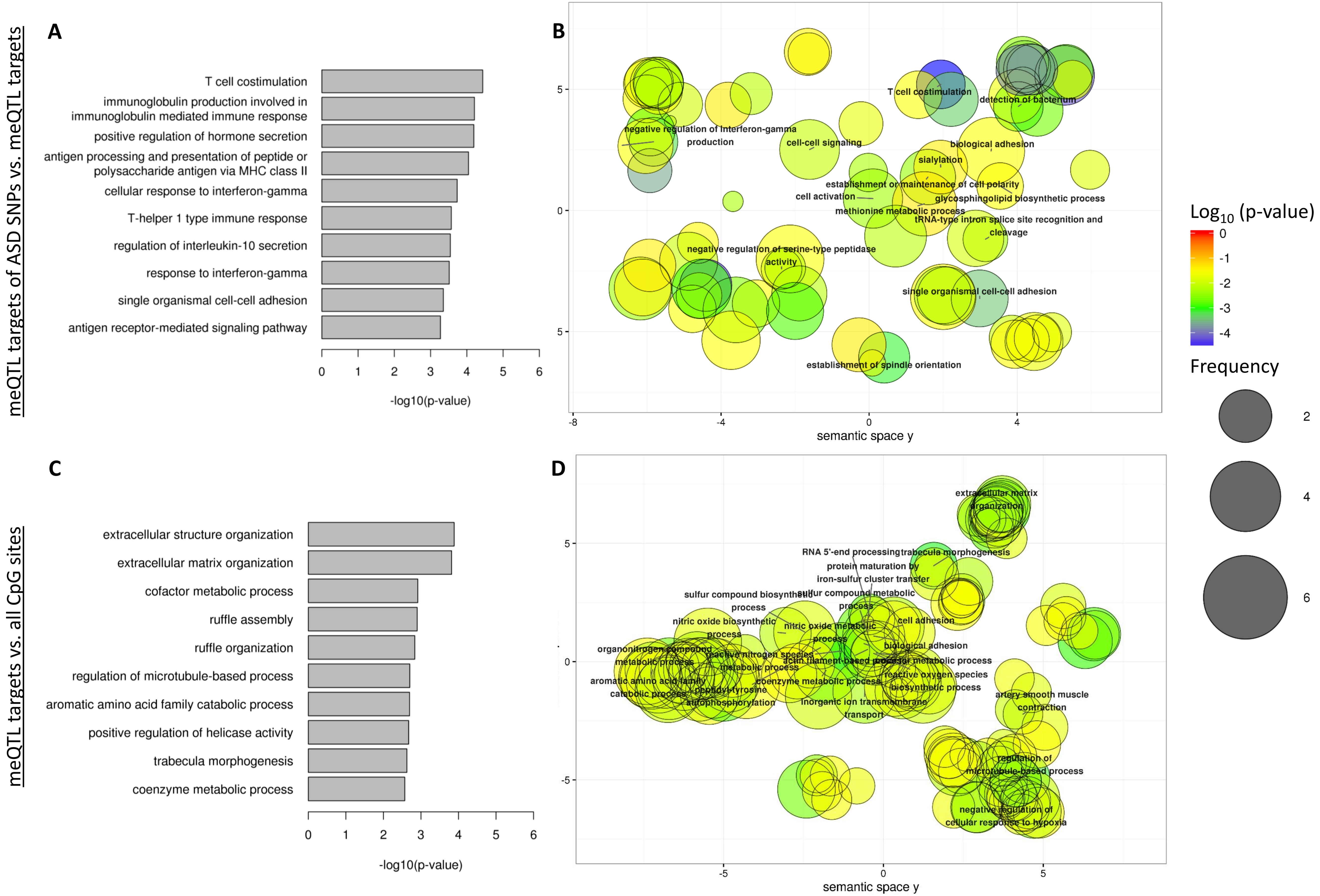

Supplementary Figure 3: **Gene Ontology enrichment analysis for meQTL targets in cord blood.** Gene Ontology (GO) enrichment analysis via the 'gometh' function in the *MissMethyl* R package. The results for the "biological processes" group were pruned to remove overlapping terms using the REVIGO software. Results shown for meQTL targets of ASD-related (PGC p-value < 1E-4) SNPs and their proxies (r^2) against a background of all meQTL targets (n = 66 vs n = 22,803; **Panels A+B**) and for meQTL targets against a background of all CpG sties tested (n = 22,803 vs n = 289,645; **Panels C+D**). meQTL targets all defined via meQTL p-value threshold = FDR 5%. **Panels A+C**) The top 10 biological process by GO enrichment p-value after REVIGO pruning. **Panel B+D**) A multi-dimensional scaling projection of the semantic similarity in nominally significant (enrichment p-value < 0.05) GO terms produced by REVIGO. Clusters are identified via labeling of the terms with both the least redundancy and highest degree of enrichment ('dispensability' value < 0.15). Color reflect degree of significance and increasing size reflects greater frequency of term in GO database.

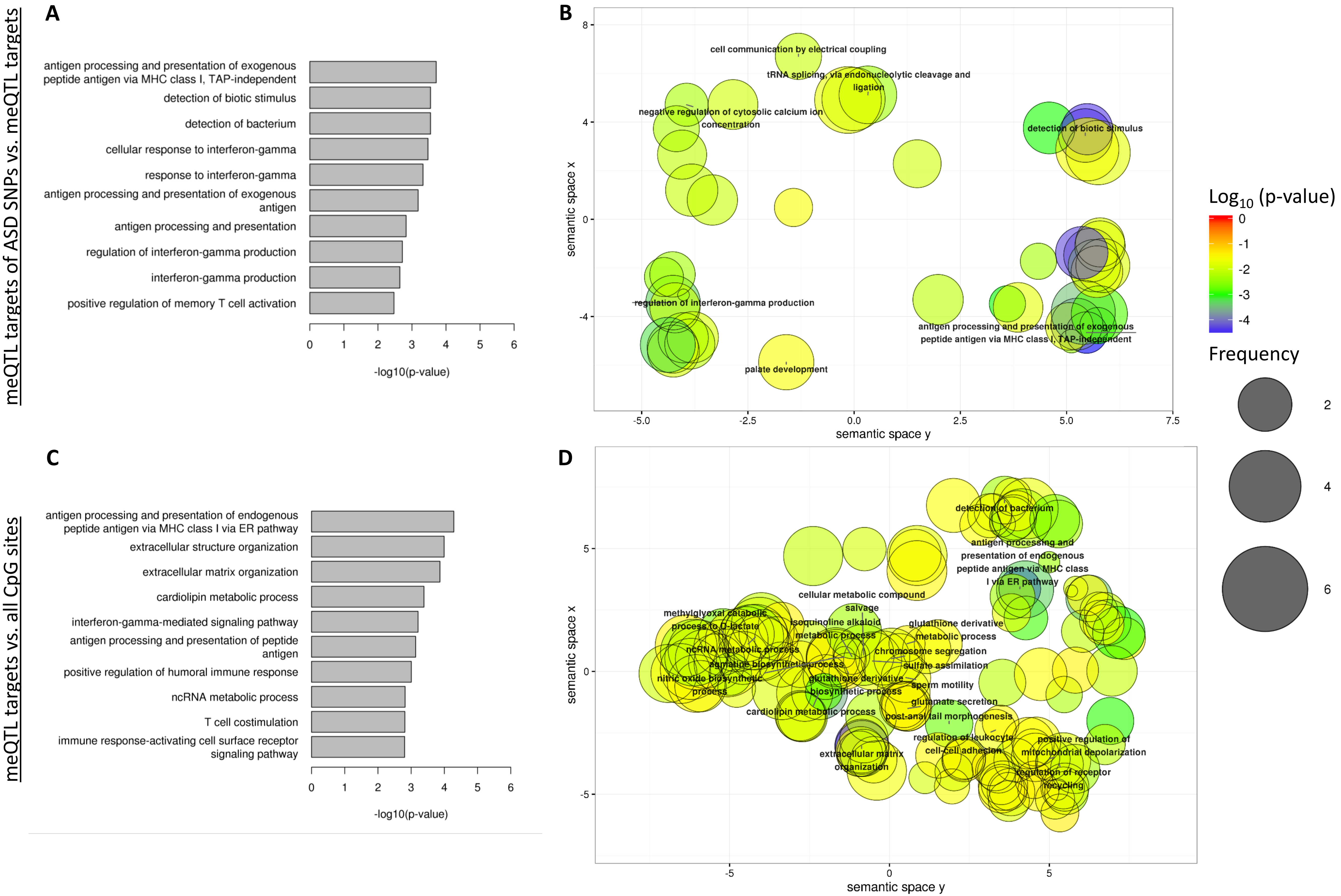

Supplementary Figure 4: **Gene Ontology enrichment analysis for meQTL targets in fetal brain.** Gene Ontology (GO) enrichment analysis via the 'gometh' function in the *MissMethyl* R package. The results for the "biological processes" group were pruned to remove overlapping terms using the REVIGO software. Results shown for meQTL targets of ASD-related (PGC p-value < 1E-4) SNPs and their proxies (r^2) against a background of all meQTL targets (n = 53 vs n = 7,863; **Panels A+B**) and for meQTL targets against a background of all CpG sties tested (n = 7,863 vs n = 314,554; **Panels C+D**). meQTL targets all defined via meQTL p-value threshold = FDR 5%. **Panels A+C**) The top 10 biological process by GO enrichment p-value after REVIGO pruning. **Panel B+D**) A multi-dimensional scaling projection of the semantic similarity in nominally significant (enrichment p-value < 0.05) GO terms produced by REVIGO. Clusters are identified via labeling of the terms with both the least redundancy and highest degree of enrichment ('dispensability' value < 0.15). Color reflect degree of significance and increasing size reflects greater frequency of term in GO database.

Supplementary Table 1: Number of genes implicated by meQTL results across tissue type

	Number of genes with meQTLs	***Number of genes with meQTL targets***	***Number of non-overlapping genes among meQTL targets***
**Fetal Brain**	7,199[a]	2,784[a]	144[a]
**Peripheral Blood**	18,454[b]<br>18,232[c]<br>17,655[d]	13,244[b]<br>12,483[c]<br>11,079[d]	597[b]<br>541[c]<br>469[d]
**Cord Blood**	15,869[b]<br>15,254[c]<br>14,015[d]	8,647[b]<br>7,876[c]<br>6,700[d]	472[b]<br>352[c]<br>226[d]
**Lung**	7,487[c]	8,087[c]	2,846[c]

[a]FDR not specified. [b]FDR = 10% [c]FDR = 5% [d]FDR = 1%

Supplementary Table 2: Summary of meQTL evidence across tissue type.

Scenario	Blood	Cord Blood	Fetal Brain	SNPs	% of Total SNPs	Independent Sites	% of Total Independent Sites
1	✓	✓	✓	125,869	4.65%	6,640	4.15%
2	✓	✓	×	407,722	15.08%	22,135	13.83%
3	✓	×	✓	30,691	1.14%	1,354	0.85%
4	✓	×	×	722,703	26.73%	42,561	26.58%
5	×	✓	✓	528	0.02%	18	0.01%
6	×	✓	×	6,299	0.23%	333	0.21%
7	×	×	✓	4,940	0.18%	237	0.15%
8	×	×	×	1,405,261	51.97%	86,821	54.23%
			**SUM**	2,704,013		160,099	

Results are shown for meQTLs associated at FDR = 5% threshold in blood and cord blood datasets and threshold of 1E-8 in fetal brain.

Only SNPs that were included in all three tissues in their respective meQTL queries are included in this analyis (n = 2,704,013).

Independent sites were constructed by grouping SNPs into bins defined by recombination hot spot data from 1000 Genomes (see Methods).

For example, scenario 1 lists that there are a total of 125,869 SNPs that are meQTLs in blood, cord blood, and fetal brain, which fall into 6,640 loci.

Supplementary Table 3: Summary of meQTL evidence for ASD-related (p < 1e-04) PGC results.

Scenario	Blood	Cord Blood	Fetal Brain	SNPs	% of Total SNPs	Independent Sites	% of Total Independent Sites
1	✓	✓	✓	5	0.46%	2	0.80%
2	✓	✓	×	74	6.76%	18	7.23%
3	✓	×	✓	0	0.00%	0	0.00%
4	✓	×	×	195	17.82%	28	11.24%
5	×	✓	✓	19	1.74%	8	3.21%
6	×	✓	×	75	6.86%	13	5.22%
7	×	×	✓	0	0.00%	0	0.00%
8	×	×	×	726	66.36%	180	72.29%
			**SUM**	1094		249	

Results are shown for meQTLs associated at FDR = 5% threshold in blood and cord blood datasets and threshold of 1E-8 in fetal brain.

All ASD-related (p< 1E-04) SNPs in PGC, regardless of if they were tested in the respective meQTL studies, are included in this analysis.

Independent sites were constructed by grouping SNPs into bins defined by recombination hot spot data from 1000 Genomes (see Methods).

For example, scenario 1 lists that there are a total of 5 SNPs that are meQTLs in blood, cord blood, and fetal brain, which fall into 2 loci.
