# Supplementary material for: Cross-tissue integration of genetic and epigenetic data offers insight into autism spectrum disorder
Source: Nat Commun. 2017 Oct 24;8:1011. doi: 10.1038/s41467-017-00868-y (PMC5654961; doi:10.1038/s41467-017-00868-y)
Supplement: Supplementary file 3 — Description of Additional Supplementary Files [file 41467_2017_868_MOESM3_ESM.pdf]

Description of Additional Supplementary Files

File Name: Supplementary Data 1
Description: Peripheral blood meQTLs identified at FDR = 5%.

File Name: Supplementary Data 2
Description: Cord blood meQTLs identified at FDR = 5%.

File Name: Supplementary Data 3
Description: Marginally significant Gene Ontology Terms post REVIGO comparing ASD-related meQTL targets to meQTL targets generally in peripheral blood.

File Name: Supplementary Data 4
Description: Marginally significant Gene Ontology Terms post REVIGO comparing ASD-related meQTL targets to meQTL targets generally in cord blood.

File Name: Supplementary Data 5
Description: Marginally significant Gene Ontology Terms post REVIGO comparing ASD-related meQTL targets to meQTL targets generally in fetal brain.

File Name: Supplementary Data 6
Description: meQTL evidence for every ASD-associated (PGC P-value < 1E-4) locus in peripheral blood, cord blood, and fetal brain.

File Name: Supplementary Data 7
Description: Enrichment Statistics comparing meQTL targets of cross-disorder PGC SNPs to meQTL targets of non cross disorder PGC associated SNPs with respect to regulatory feature overlap.

File Name: Supplementary Data 8
Description: Enrichment Statistics comparing meQTL targets to non-meQTL targets with respect to regulatory feature overlap.
